# Stress-induced premature senescence in high five cell cultures: a principal factor in cell-density effects

**DOI:** 10.1186/s40643-024-00824-x

**Published:** 2024-11-25

**Authors:** Rui Min, Dahe Zhang, Mingzhe He, Jingyuan Chen, Xiaoping Yi, Yingping Zhuang

**Affiliations:** 1 State Key Laboratory of Bioreactor Engineering, East China University of Science and Technology (ECUST), 130 Meilong Rd, Shanghai, 200237 China; 2 Womei Biology Company, Limited, Suzhou, China

**Keywords:** Cell density effect, Transcriptome, Proteome, Time-series analysis, Stress-induced premature senescence, Mitochondrial dysfunction

## Abstract

The Baculovirus Expression Vector System (BEVS) is highly valued in vaccine development, protein engineering, and drug metabolism research due to its biosafety, operational convenience, rapid scalability, and capacity for self-assembling virus-like particles. However, increasing cell density at the time of inoculation severely compromises the production capacity of BEVS, resulting in the “cell density effect”. This study aimed to explore the mechanisms of the cell density effect through time-series analysis of transcriptomes and proteomes, with the goal of overcoming or alleviating the decline in productivity caused by increased cell density. The dynamic analysis of the omics of High Five cells under different CCI (cell density at infection) conditions showed that the impact of the “cell density effect” increased over time, particularly affecting genetic information processing, error repair, protein expression regulation, and material energy metabolism. Omics analysis of the growth stage of High Five cells showed that after 36 h of culture (cell density of about 1 × 10^6^ cells/mL), the expression of ribosome-related proteins decreased, resulting in a rapid decrease in protein synthesis capacity, which was a key indicator of cell aging. Senescence verification experiments showed that cells began to show obvious early aging characteristics after 36 h, resulting in a decrease in the host cell’s ability to resist stress. Overexpression and siRNA inhibition studies showed that the *ndufa12* gene was a potential regulatory target for restricting the “cell density effect”. Our results suggested that stress-induced premature senescence in High Five cell cultures, resulting in reduced energy metabolism and protein synthesis capabilities, was a critical factor contributing to cell density effects, and ultimately affecting virus production. In conclusion, this study provided new insights into managing virus production limitations due to cell density effects and offered innovative strategies to mitigate the adverse effects of cellular aging in biomanufacturing technologies.

**Graphical abstract:**

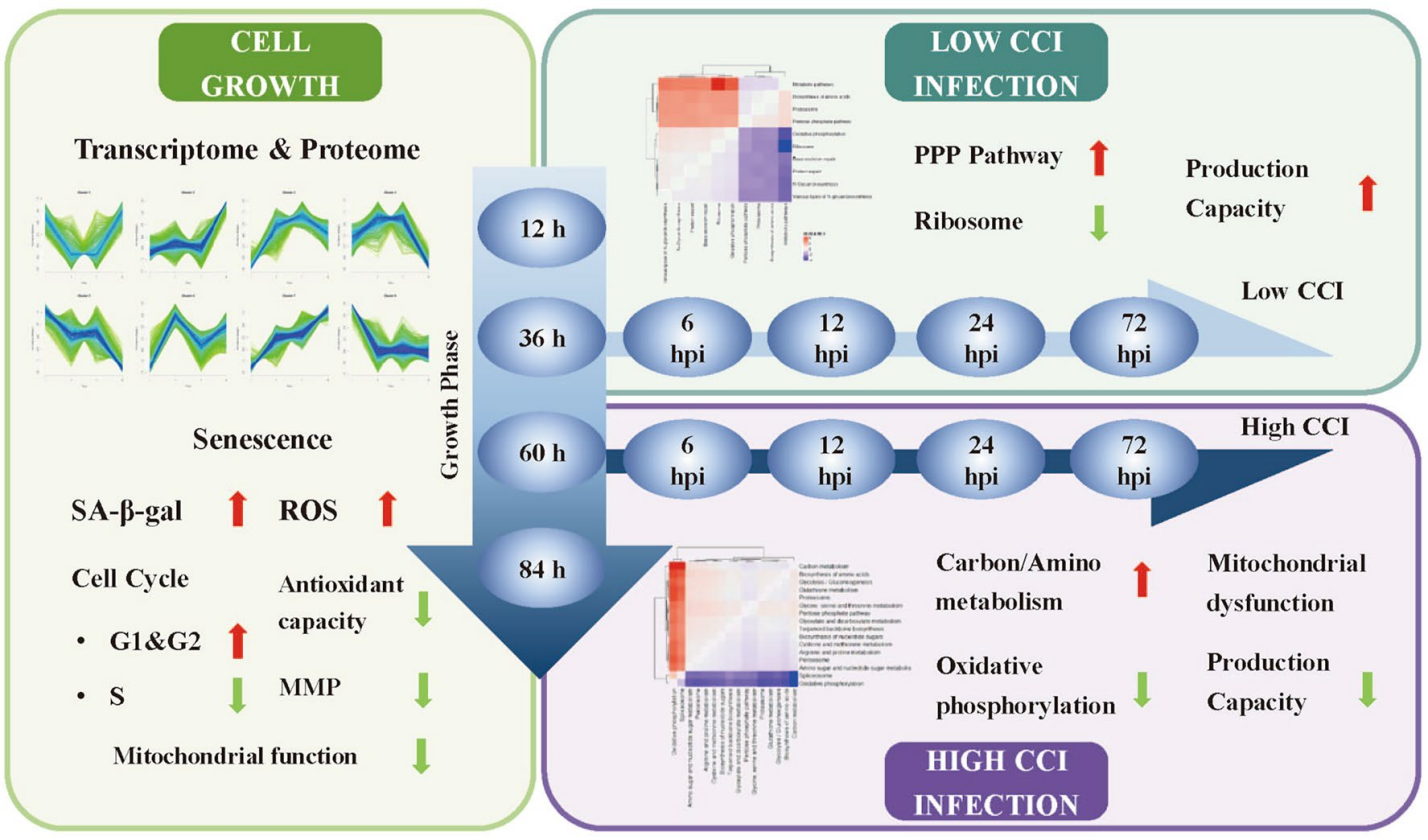

**Supplementary Information:**

The online version contains supplementary material available at 10.1186/s40643-024-00824-x

## Introduction

Baculoviruses are enveloped viruses with circular double-stranded DNA genomes (80–180 kb) that specifically infect invertebrates, particularly Lepidoptera insects (Clem and Passarelli 2013). The Baculovirus Expression Vector System (BEVS) leverages the natural ability of baculoviruses to infect insect cells and produce large quantities of proteins by utilizing the very late promoters, such as polyhedrin (polh) and p10. These promoters exhibit high expression levels late in the infection cycle, allowing the baculovirus to efficiently use the host cell machinery for the production of recombinant proteins. The versatility of BEVS comes from its ability to accommodate large inserts of foreign DNA, facilitating the expression of a wide range of proteins, including complex eukaryotic proteins requiring post-translational modifications (e.g., glycosylation, phosphorylation) (Kost et al. 2005). Since the first use of BEVS for the β-interferon production in 1983 (Smith et al. 1983), the baculovirus expression system has undergone rapid development over the past four decades and has become one of the most prominent protein expression systems (Contreras-Gomez et al. 2014; Maiorella et al. 1988; van Oers et al. 2015). Numerous BEVS-produced vaccines are now available, including human vaccines (Cervarix, Flublok (Yang 2013), Flublok Quadrivalent), therapeutic proteins for humans (Provenge and Glybera), and veterinary vaccines (Porcilis Pesti, BAYOVAC CSF E2, Circumvent PCV, Ingelvac CircoFLEX, and Porcilis PCV) (Felberbaum 2015).

High Five cells, derived from the ovarian tissue of the cabbage looper *Trichoplusia ni*, are one of the most widely used insect cell lines in BEVS. High Five cells have several advantages over other insect cell lines, such as Sf9 and Sf21, including higher expression levels of recombinant proteins, greater tolerance to nutrient deprivation, and a capability to grow to higher cell densities in serum-free medium (Wickham et al. 1992).

In the realm of BEVS, a significant production constraint known as the “cell density effect” emerges. This phenomenon is characterized by a sharp decline in the specific productivity as the cell density increases (Hink et al. 1977; Stockdale and Gardiner 1977; Wood et al. 1982). Wickham et al. (1992) assessed the productivity and viral infectivity of eight different insect cell lines, identifying the cell density-dependent suppression of specific protein and virus yields. Studies have shown that high cell density significantly reduced BEVS productivity when the Cell Concentration at Infection (CCI) exceeded 1 × 10^6^ cells/mL (Chico and Jäger 2000; Huynh et al. 2013). Various speculated causes included nutrient limitations, precursor depletion, accumulation of metabolic byproducts, cell contact inhibition, and growth factor depletion (Caron et al. 1990; Taticek and Shuler 1997). However, these supplementation strategies only alleviate the cell density effect to a certain extent. The fed-batch cultures of insect cells can achieve cell densities of tens of millions per milliliter, yet in reality, cell density effects limit virus inoculation density to below 3 × 10^6^ cells/mL. Numerous studies have confirmed that the deterioration in protein yields with increased cell density cannot be simply attributed to the consumption of essential nutrients or the accumulation of inhibitory byproducts (Bernal et al. 2009; Carinhas et al. 2010; Huynh et al. 2013). Exploring the microphysiological changes in cells, particularly the transition from the exponential phase to the stationary phase, might offer an effective approach to investigating the mechanisms underlying the cell density effect (Yin and Redovich 2018).

The advent of omics technologies (genomics, transcriptomics, proteomics, and metabolomics) has enabled researchers to delve into the complex molecular changes triggered by cell density and the profound impact of these effects on virus production. Lucy LeBlanc et al. (2022) used transcriptome sequencing technology to study the effects of cell seeding density on mouse embryonic stem cells (mESCs) and discovered important regulatory factors that directly regulated cell density-sensitive genes during spontaneous differentiation. Lavado-Garcia et al. (2020) used quantitative proteomics to study HEK-293 cells during virus-like particle (VLP) production, discovering that downregulated lipid and protein synthesis limited virus transfection and expression. Carinhas et al. (2011) compared the proteomic responses of host cells infected at low cell density (1.5−2 × 10^6^ cells/mL) and high cell density (4−4.5 × 10^6^ cells/mL), finding differences mainly related to substance energy metabolism, oxidative stress, and protein processing pathways. Due to limited annotation information, this study identified only 648 usable protein information.

A single-omics approach may provide only a partial understanding of the subject, making it challenging to accurately explore the precise regulation of cells and their broad responses after viral infection. In this study, we employed a multi-omics approach to reveal the molecular mechanisms underlying the cell density effect, specifically the interaction between cell growth and viral reproduction, and to identify molecular targets to mitigate the cell density effect.

## Method

### Cell culture and virus infection

*Trichoplusia ni* derived BTI-Tn-5B1-4 (High Five) cell line was cultured in 1L shake flasks with a working volume of 200 mL of HF-SFM medium (Womei Biotech Co., Ltd., Suzhou, China), under conditions of 27 °C and 110 rpm. High Five cells were inoculated at a density of 3 × 10^5^ cells/mL, and baculovirus was inoculated at 36 h, 60 h, and 84 h, corresponding to CCI1 (0.5–1 × 10^6 cells/mL), CCI3 (2–3 × 10^6 cells/mL), and CCI5 (4–5 × 10^6 cells/mL), respectively. Viable cell density, viability, and diameter were measured using a Countstar Bio (Ruiyu Biotech Co., Ltd., Shanghai, China).

*Autographa californica multicapsid nucleopolyhedrovirus* was used as the expression vector for recombinant *Porcine Circovirus* Type 2 (PCV2). Virus amplification was performed by infecting *Spodoptera frugiperda* derived Sf9 cells. Quantification of infectious baculovirus particles was performed following TCID50 assay as described in Roldao et al. (2009), with each sample tested in duplicate. Total PCV2 titer was quantified in cell suspension samples disrupted by an XO-650 ultrasonic cell disruptor (Xianou IM Co., Ltd., Nanjing, China) at 72 h post-infection (hpi). The disruption was set at 30% power for 30 min, with an operational time of 2 s and an interval of 5 s. Centrifuge the disrupted sample at 13,000 rpm for 30 min and quantify the PCV2 titers using a sandwich ELISA kit (INGENASA, Spain), with each sample tested in triplicate. Transcriptome and proteome sampling included cell culture stages (12 h, 36 h, 60 h, 84 h) and virus-infected stages at CCI1 and CCI3 (6 hpi, 12 hpi, 24 hpi, 72 hpi), with three biological replicates. The MOI of each CCI condition was set to 2 to maintain infection consistency.

### RNA sequencing and data analysis

For transcriptome, 5 × 10^6^ cells were collected, and centrifuged at 1000 rpm. Cell pellet was washed with PBS buffer twice and suspended in 1 mL of Trizol (Tiangen Biotech Co., Ltd., Beijing, China), and frozen in liquid nitrogen before being stored at − 80 °C. RNA extraction and transcriptome sequencing were performed at Biomarker Technologies Corporation (Beijing, China), with assembly and annotation completed on the BMK Cloud platform.

To characterize the global expression profiles, self-organizing map (SOM) analysis was performed using the kohonen package in R (Wehrens and Buydens 2007). Principal Component Analysis (PCA) was used to analyze correlations to explore sample-to-sample relationships. Global differential expression analysis under different CCI conditions was performed according to previous study (Schmidt et al. 2018). Briefly, three methods based on R packages were used: (1) a DESeq2 (Love et al. 2014) generalized linear model of the negative binomial family with time as a discrete factor; (2) a DESeq2 generalized linear model of the negative binomial family with time represented as a natural cubic spline; and (3) a maSigPro (Nueda et al. 2014) cubic polynomial regression. The model design formula considered time factors, CCI conditions, and their interactions. To characterize transcriptional expression differences during the cell culture stages, the mfuzz package was used to cluster genes with similar expression profiles (Kumar and Futschik 2007). Local differential expression analysis was performed by DESeq2 (Love et al. 2014), and differentially expressed genes (DEGs) were identified with absolute log2 (fold change) > 1 and adjusted *p* value < 0.05. KEGG/GO enrichment analysis and Gene Set Enrichment Analysis (GSEA) were performed using Clusterprofiler (Yu et al. 2012), with adjusted *p* value < 0.05 considered significant.

### Protein extraction and mass spectrometry sample preparation

For proteome, 1 × 10^6^ cells were collected, and centrifuged at 1000 rpm. Cell pellet was washed with PBS buffer twice and frozen in liquid nitrogen before being stored at − 80 °C. 0.1 ml of homemade lysis buffer was added to each sample (Ross et al. 2002). After that, the samples were homogenized at 4 °C and then incubated for 30 min on ice. Protein concentration was quantified by standard BCA assay. The lysates were centrifuged at 20,000 g for 30 min at 4 °C and protein concentration was determined by standard BCA assay.

For each sample, 20 μg of protein was denatured with 6 M urea and reduced using 5 mM tris (2-carboxyethyl) phosphine (TCEP). Proteins were then alkylated with 6.25 mM iodoacetamide (IAA) for 30 min at room temperature in the dark. The mixture was diluted with 6 vol of 50 mM ammonium bicarbonate and digested with sequence-modified trypsin (Promega, Madison, WI) at an enzyme-to-substrate ratio of 1:30 (w/w) for 12 h at 37 °C. The pH of each sample was then adjusted to 2–3. Finally, the acidified mixture was purified using a 96-well C-18 cartridge plate (ThermoFisher, USA) and dried under vacuum.

The dried peptides were dissolved in 0.1% formic acid (0.5 μg/μL) and labeded using TMT10-plex reagent according to the manufacturer's protocol (Thermo Fisher Scientific, USA). Four 10-plexes were used, and an internal standard containing a mix of protein from all samples was used as one TMT tag in each TMT set to enable relative quantification between TMT sets.

### Liquid chromatography tandem mass spectrometry (LC–MS/MS) analysis

Proteomic sequencing was performed at Shanghai Center for Systems Biomedicine, Shanghai Jiao Tong University, Shanghai, China. Before analysis on an Orbitrap Fusion mass spectrometer (Thermo Fisher Scientific, USA), peptides were separated using a Dionex Ultimate 3000 (Thermo Fisher Scientific, USA). The labeled peptide mixtures from each TMT group were delivered to an analytical column (Dikma, inspire C18, 3 µm, Canada, 150 mm × 75 µm, self-packed). At a flow rate of 0.3 µL/minute, Buffer A (0.1% formic acid in H2O) and Buffer B (0.1% formic acid in 80% ACN) were used to run a gradient from 3 to 7% Buffer B for 5 min, 7–22% for 50 min, 22–35% for 12 min, 35–80% for 1 min, and finally 80% for 8 min. The fractions were collected every 2 min and finally combined into 12 pools.

The Orbitrap Fusion mass spectrometer operated in data-dependent acquisition (DDA) mode. Full scan MS spectra (350–1550 m/z) were acquired in the Orbitrap at 60,000 resolution with a target value of 200,000 for a maximum time of 100 ms. Tandem mass spectra were recorded for maximum 3 s by higher energy collision induced dissociation at a normalized collision energy of 30% in the orbitrap. Dynamic exclusion was enabled with one repeat count in 60 s exclusion time. All Orbitrap data were searched using SequestHT under the Proteome Discoverer 1.4 software platform (Thermo Fisher Scientific), filtered to a 1% FDR. TMT reporter ion ratios were median normalized within sample median (ratio R against internal standard) using PSMs from peptides unique to each gene symbol. Consider proteins detected in at least two out of three replicates for each protein.

### Proteomics data analysis

To characterize the global expression profiles, limma (Ritchie et al. 2015) R package was used to model the group differences at baseline, time point differences, and group-specific differences over time (interaction term), similar to the method used for detecting DEGs. Two methods were applied based on whether B-splines fitting (with 3 degrees of freedom) was used or not. A protein was considered differentially expressed with FDR < 0.05 in either method. To assess the contribution of time and CCI condition factors to the proteomic data, the MetStat (Dorscheidt 2013) package in R was used for ANOVA-simultaneous component analysis (ASCA) on the log2-transformed means of all samples from CCI1 and CCI3. The screening methods and permutation tests were consistent with those described by Wanamaker et al. (2020). The mfuzz (Kumar and Futschik 2007) package was used to cluster genes with similar protein expression levels during cell culture. For differential gene analysis, proteins were identified as differentially expressed with a t-test fold change > 1.2 and padj < 0.05. Gene function and pathway enrichment analyses for the differentially expressed proteins were performed using the ClusterProfiler (Yu et al. 2012) package, based on the Gene Ontology (GO) database (http://geneontology.org/) and the KEGG database (https://www.kegg.jp/kegg/pathway.html). Gene Set Enrichment Analysis (GSEA) and Differential Gene Set Enrichment Analysis (DGSEA) were performed using the ClusterProfiler package to evaluate the relative enrichment levels between different pathways (Joly et al. 2021).

### Cell senescence analysis

For cell cycle detection, samples were processed using a cell cycle detection kit (Beyotime Biotech Co., Ltd., Shanghai, China). β-galactosidase activity was assessed using the Cell Meter Cellular Senescence Activity Assay Kit (ATT Bioquest, USA). To avoid potential damage to insect cells at 37 °C, the incubation temperature for the fluorescent probes in both kits was controlled at 27 °C. Apart from the temperature adjustment, all procedures followed the standard protocols provided with the kits. Detection was performed on a CytoFLEX flow cytometer (Beckman Coulter, USA).

### Mitochondrial respiratory capacity and membrane potential analysis

Mitochondrial function was measured using the Oxygraph-2 k mitochondrial function analyzer (Oroboros, Innsbruck, Austria), assessing the cellular oxygen consumption rate (OCR). Basal respiration was measured by diluting cell suspension to 5 × 10^5^ cells/mL and adding it to the test chamber, recorded as Routine. ATP synthase was inhibited by 1 μL 4 mg/ml oligomycin (Sigma-Aldrich, San Luis, MO, USA), recorded as Leak. The oxygen consumption level reached the maximum, with the supplement of 1 μL 1 mM FCCP (Carbonylcyanide p-trifluoromethoxyphenylhydrazone, Abcam, Cambridge, UK), recorded as electron transfer system (ETS). 1 mM rotenone (Abcam, Cambridge, UK) and 1 mM antimycin A (Abcam, Cambridge, UK) were used to inhibit mitochondrial complex I/III, recorded as residual oxygen consumption (ROX). The ATP production was calculated using the difference between Routine and Leak respiration, and the spare respiratory capacity (SRC) was determined using the difference between ETS and ROX respiration.

The mitochondrial membrane potential was measured using a mitochondrial membrane potential assay kit (Beyotime Biotech Co., Ltd., China) according to the manufacturer’s instructions. Fluorescence intensities of JC-1 monomers (Ex/Em: 485/530 nm) and aggregates (Ex/Em: 485/590 nm) were detected using a multifunctional microplate reader (Thermo Fisher Scientific, USA). Mitochondrial membrane potential was assessed by measuring the ratio of monomeric and aggregated fluorescence intensities. The incubation temperature for the fluorescent probes was maintained at 27 °C. Measurements were performed in triplicate for each sampling point.

### Redox state detection

Intracellular ROS levels were measured using a DCFH-DA ROS detection kit (Jiancheng Biotech Co., Ltd., Nanjing, China) according to the manufacturer’s protocol, with fluorescence intensity recorded at an excitation wavelength of 488 nm and an emission wavelength of 525 nm. Comparisons of ROS levels were made directly between groups based on relative fluorescence units (RFUs). Intracellular reduced glutathione concentration was assessed using the QuantiChrom™ Glutathione (GSH) Assay Kit (Bioassay, USA) with absorbance measured at OD 412 nm. A standard curve ranging from 0 to 100 µM was used for quantification. Intracellular NADH concentration was determined using the EnzyChrom™ NAD/NADH Assay Kit (Bioassay, USA) with absorbance measured at OD 565 nm. All measurements were performed on a fluorescence microplate reader (Thermo Fisher Scientific, USA). A standard curve ranging from 0 to 10 µM was used for quantification. The final results for GSH and NADH were expressed as the ratio of the amount of GSH or ROS to the number of cells collected. Measurements were performed in triplicate for each sampling point.

### qPCR detection

Total RNA was extracted using TRIeasy™ Total RNA Extraction Reagent (10606ES60, Yeasen, Shanghai, CN), and RNA concentration and purity were measured by NanoDrop spectrophotometer (Thermo Fisher Scientific, USA). Reverse transcription was performed by reverse transcription kit (Thermo Fisher Scientific, USA). Quantitative polymerase chain reactions were performed by real-time PCR enzymes (Yeasen Biotech Co., Ltd., Shanghai, CN) and detected on real-time fluorescence PCR system (Thermo Fisher Scientific, USA). Gene expression levels were normalized using the *ACT* gene encoding for acting as an internal control. Each sample was measured in three parallel replicates. The forward and reverse primer sequences of these genes are shown in Table S1.

### siRNA analysis

siRNAs targeting *qcr6*, *ndufa12*, and negative control siRNAs, were purchased from Genma Pharmaceutical Technology Co., Ltd.(Shanghai, China). To avoid off-target effects, two different siRNAs for each gene were used, and the sequence with the best interference effect was selected (Table S2). The siRNA sequences are shown in Table S2. The High Five cells were seeded in 6-well plates at a density of 100,000/well, with 2 mL of cell suspension per well. After 24 h, the cells were transfected with 200 nM siRNA targeting *qcr6*, *ndufa12*, and non-targeting controls by Lipofectamine 2000 (Thermo Fisher Scientific, USA) in HF-SFM medium (Womei Biotech Co., Ltd., Suzhou, China). After 48 h, adherent cells were transferred to 50 mL shaker flasks for suspension culture. Samples were taken for mitochondrial function and membrane potential detection when cell density reached 1 × 10^6^ cells/mL. The cultivation was infected at an MOI1, and total PCV2 titers were measured at 72 hpi.

### Overexpression analysis

Primers were designed to amplify the QCR6 and NDUFA12 genes. The pIB vector was digested with BamhI endonucleases (TAKARA, Japan). The target genes were ligated into the vector using a seamless cloning kit (Beyotime Biotech Co., Ltd., China). High Five cells were seeded in 6-well plates for 24 h and then transfected with 2000 ng of recombinant plasmid for each experiment, supplemented with appropriate concentrations of non-target controls (original pIB plasmid). Transfection was performed by Lipofectamine 2000 reagent (Thermo Fisher Scientific, USA) in HF-SFM medium (Womei Biotech Co., Ltd., Suzhou, China). 20 μg/mL blasticidin (Beyotime Biotech Co., Ltd., China) was added to the medium 1 day post-transfection. After 48 h, adherent cells were transferred to 50 mL shaker flasks for suspension culture. Samples were taken for mitochondrial function and membrane potential detection when cell density reached 3 × 10^6^ cells/mL. Cells were infected at an MOI1 and total PCV2 titers were measured at 72 hpi. The forward and reverse primer sequences of these genes are shown in Table S3.

### Statistical analysis

All data are expressed as mean ± standard deviation (SD). Errors and significance were analyzed by GraphPad Prism 8.0. T test was used to compare the two groups. *p* < 0.05 (*) was considered statistically significant, *p* < 0.01 (**) was considered highly significant, *p* < 0.001 (***) and *p* < 0.0001 (****) was considered extremely significant.

## Results

### Impact of cell density effect on the production capacity of PCV2 in high five cells

**Fig. 1:**
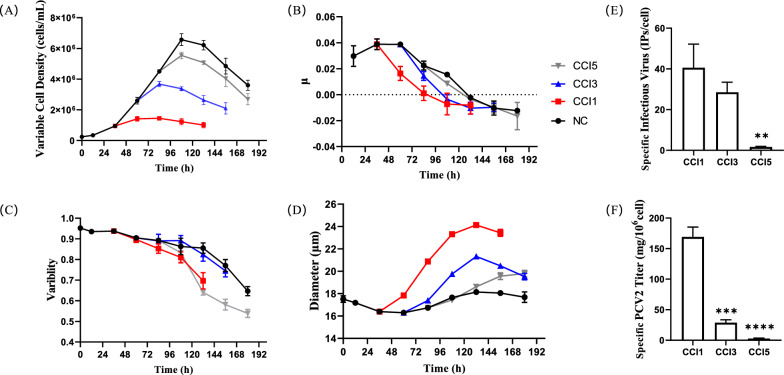
The Impact of Cell Density Effect on Virus Infection and PCV2 Production under different CCI conditions. **A** Viable cell density; **B** Specific growth rate of cells; **C** Cell viability; **D** Changes in cell diameter; **E** Quantitation of infectious virus particles per cell at 72 hpi; **F** Specific production capacity of PCV2 at 72 hpi

To investigate the cell density effect, we recorded the viable cell density, specific growth rate, viability, and cell diameter during the cell culture and viral infection under different Cell Concentration at Infection (CCI). Baculovirus infection suppressed cell growth, leading to a decrease in viable cell density and a marked reduction in specific growth rate and viability (Fig. 1a–c). However, the increase in CCI hindered the suppression effect of viral infection on cell growth. In the CCI3 and CCI5 groups, the cells exhibited significant proliferation post-infection, whereas cell growth in the CCI1 group stopped immediately. Additionally, virus infection caused an increase in cell diameter, which negatively correlated with CCI (Fig. 1d). In the CCI1 group, the cell diameter reached up to 23 μm at 96 h post-infection (hpi), whereas the maximum cell diameter in the CCI5 group was only 20 μm, indicating that virus-induced cytopathic effects (such as swelling and fusion) were mitigated with higher inoculation densities. These results suggested that with increasing CCI, the cells exhibit a delayed response to viral infection.

The quantification of infectious baculovirus particles reflected the state of viral infection, which directly affected the yield of the product in BEVS. In this study, cells were infected at an MOI of 1 under CCI1, CCI3, and CCI5. At 72 hpi, the number of extracellular infectious baculovirus particles was 40.58 ± 11.57, 28.54 ± 4.94, and 1.65 ± 0.28 IPs/cell, respectively (Fig. 1e). At this time, the specific total PCV2 productivity was 169.10 ± 16.09, 28.76 ± 4.88, and 2.81 ± 0.69 mg/10^6^ cells, respectively (Fig. 1f). Similar to other studies (Chico and Jäger 2000), when High Five cells are inoculated with baculovirus at cell densities exceeding 1 × 10^6^ cells/mL, a significant cell density effect occurs, severely hindering baculovirus replication and PCV2 production capacity.

### Global time-series analysis based on transcriptomic and proteomics

To explore the intrinsic mechanisms of the cell density effect, we performed a time-series analysis of transcriptomic and proteomics for the CCI1 and CCI3, including four time points during the cell culture stage (12 h, 36 h, 60 h, 84 h) and five time points during the virus infection stage (0 hpi, 6 hpi, 12 hpi, 24 hpi, 72 hpi). The 36 h and 60 h cell culture stages corresponded to the 0 hpi of virus infection for the CCI1 and CCI3, respectively. Through transcriptomic sequencing, a total of 12,505 genes were identified across all samples. With our mass spectrometry-based proteomics approach, proteins that were detected in at least two of the three parallels at each sampling point were considered credible, and finally 3232 proteins were identified.

**Fig. 2:**
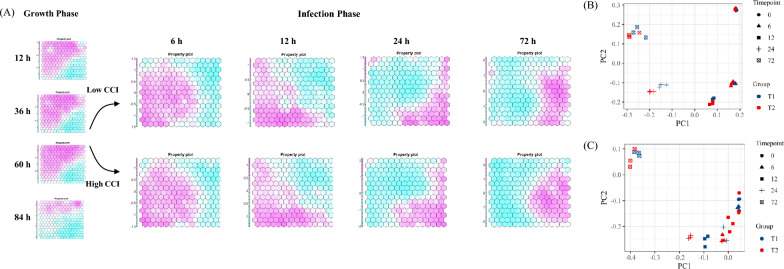
**A** Global sample Self-Organizing Map (SOM) analysis; **B** PCA of the transcriptome; **C** PCA of the proteome

Unsupervised clustering of the transcriptome was performed using the Self-Organizing Map (SOM) method, presenting the global dynamics induced by the “cell density effect” through a polarized topological structure (Fig. 2a). The results indicated that during the cell culture stages of 12 h, 36 h, and 60 h, overall transcriptomic changes were minor, and genes related to the upper left of the topology map were highly expressed. However, most genes were downregulated at 84 h, indicating an overall transcriptional capacity deteriorating in the later stages of cell culture. Viral infection, especially at 24 hpi, observably suppressed the genes with high expression during cell culture and led to a gradual increase in the expression of genes in the lower right quadrant of the topology map. Principal Component Analysis (PCA) was used to extract the relationships between CCI1 and CCI3 groups (Fig. 2b and c). For the transcriptome, PC1 and PC2 primarily distinguished the control groups in a counterclockwise direction by the time factor, with differences between CCI groups increasing over time. For the proteome, time and CCI factors were clearly distinguished under the principal components PC1 and PC2, displaying more complex interactive effects between the two factors. These results indicated that the CCI1 and CCI3 groups exhibited similar dynamic trends in the early stages of baculovirus infection, with differences between the two groups increasing over time.

**Fig. 3:**
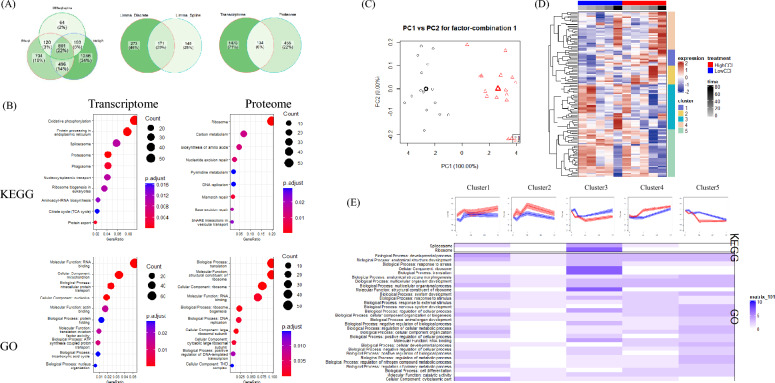
Comparison of the differences in transcriptome and proteome over time during infection under different CCI conditions; **A** Venn diagram for temporal analysis of differential genes and proteins; **B** KEGG and GO enrichment analysis of differential genes and proteins; **C** Score plot for the CCI factor derived from ASCA analysis of all protein abundances; **D** Protein abundances of the top 259 proteins most impacted by the CCI factor; **E** Temporal abundance patterns of 259 proteins affected by time, based on five evolutionary branches

To directly compare the temporal changes of viral infection under different CCIs, various time-series analysis methods to identify gene/protein with differential dynamic trends. The detailed temporal analysis methods can be found in the Methods section. In total, 1610 differential genes and 589 differential proteins were identified. Venn diagrams of differential genes and proteins showed that only 8% of the genes/proteins overlapped (Fig. 3a). Baculovirus infection produces profound effects on the genetic regulatory functions of the host cell, including post-transcriptional modifications, protein translation and degradation, and genomic level regulation. Virus infection may cause extensive degradation of host mRNA (Narayanan and Makino 2013). Early in the infection, the baculovirus modulates the expression of host genes through various mechanisms, including direct interference with the host transcription machinery by viral proteins (Iwanaga et al. 2007). During the infection, certain host cell protein levels decrease, possibly regulated by viral miRNAs (Nayyar et al. 2017). These factors were supposed to exacerbate the inherent differences between the host cell transcriptome and proteome, underscoring the significance of integrative omics analysis. Enrichment analysis of differential pathways such as oxidative phosphorylation, ER protein processing, spliceosome, proteasome, and autophagy in the KEGG pathways, and RNA binding, mitochondria, intracellular protein transport, and nucleolus in the GO terms (Fig. 3b). Proteomic differences were mainly enriched in ribosome, carbon metabolism, amino acid synthesis in KEGG pathways, and transcription, translation, and ribosome in GO terms (Fig. 3b).

The differences in the transcriptome and proteome between the CCI1 and CCI3 groups were a result of the interplay between cell growth and virus infection. To further distinguish the complex interactive effects observed in proteome (Fig. 2c), ANOVA Simultaneous Component Analysis (ASCA) was performed to separate the expression patterns by time, CCI, and their interaction. The results showed that time accounted for 76.41% of protein abundance changes, while CCI accounted for 3.03%. Permutation tests validating the large variable effects of ASCA indicated that only time had a significant impact on protein abundance (Table 1). Nevertheless, the minor differences induced by CCI amplified over time, ultimately affecting the production of PCV2 (Fig. 2). When examining the CCI effect components partitioned by ASCA, PC1 could distinguish differences induced by CCI effects at each sampling point (Fig. 3c). Following the same selection scheme (Wanamaker et al. 2020), a total of 259 proteins contributed the most to abundance pattern differences across CCI when proteins were filtered. Hierarchical clustering of these protein abundance patterns revealed five distinct evolutionary branches, which had different expression trends throughout the time (Fig. 3d and e). In Cluster 1, the CCI3 group exhibited higher expression levels than the CCI1 group throughout the process. KEGG and GO enrichment analysis results showed that these genes are associated with developmental processes and intracellular components. In Cluster 3, the CCI1 group exhibited higher expression levels than the CCI3 group throughout the process. KEGG and GO enrichment analysis results showed that these genes are related to spliceosome, ribosome, and translation, which indicate a stronger capacity for genetic information processing. The replication of the baculovirus genome was initiated by exploiting enhancers and transcription activators in the transcription machinery of host cells (Saxena et al. 2018). The functional capability of the host cell may affect the translation efficiency of viral proteins, thereby affecting the replication and expression of late target genes. This impact might be cascading, as the baculovirus underwent secondary infection after completing an infection cycle in the production system.

**Table 1:** Contributions of experimental factors to the variation in ASCA partitioned data and permutation validation test results

Factor	Variation (%)	Permutation test (*p* value)
CCI	3.03	0.100
Time	76.41	0.001
CCI: Time	20.56	0.278

## Cellular senescence leading to decline in ribosomal function in high five cells

**Fig. 4:**
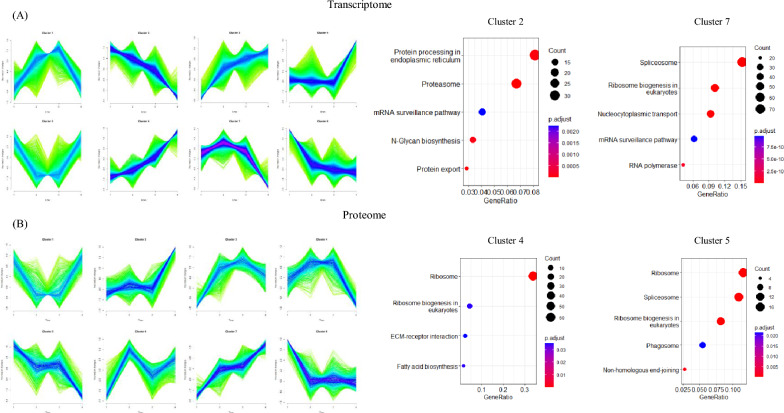
**A** Cluster analysis of transcriptomes and proteomes during cell culture; **B** Enrichment analysis of specific clusters

Considering that the differences in the transcriptome and proteome of different CCI groups were the interactive result of cell growth and viral infection, we analyzed the dynamic changes in the cell culture stage to determine the causes of the weakened genetic information processing capacity. Temporal expression clustering was performed using the mfuzz toolkit, resulting in the identification of eight transcriptome clusters and eight proteome clusters with distinct profiles (Fig. 4a). For the transcriptome, Cluster 2 showed a decreasing trend at all stages of cell culture, while Cluster 7 peaked at 36 h and rapidly declined at 60 and 84 h. Cluster 2 was enriched in proteins involved in endoplasmic reticulum processing and the proteasome, while Cluster 7 was enriched in the spliceosome, ribosome biogenesis, and nucleocytoplasmic transport. In the proteome, Cluster 4 and Cluster 5 remained consistent at 36 and 60 h but rapidly declined at 84 h. Cluster 4 was primarily enriched in ribosome, while Cluster 5 was enriched in ribosome, spliceosome, and ribosome biogenesis pathways. The results indicated that, in the absence of viral infection, the protein synthesis capacity of host cell rapidly declined in the later stages of culture. Changes in ribosome biogenesis and a decrease in overall protein synthesis rates are characteristics of the aging process in many organisms (Anisimova et al. 2018). Therefore, this comprehensive decline in cellular translation machinery was hypothesized to be associated with senescence.

## High cell density infection leads to rapid downregulation of oxidative phosphorylation pathway

**Fig. 5:**
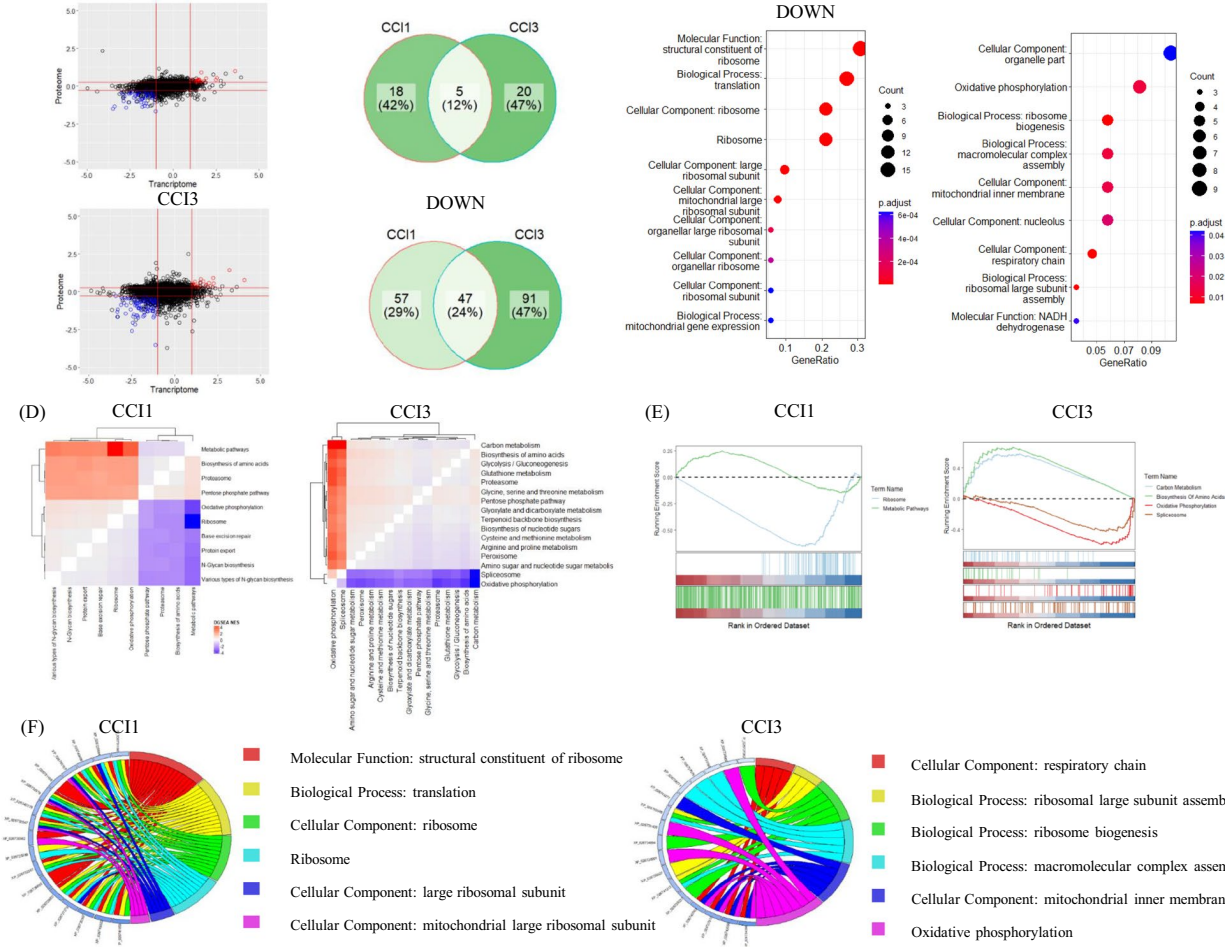
Integrative analysis of the transcriptome and proteome of High Five cells infected under different CCI conditions at 24 hpi. **A** Scatterplot of fold changes in the transcriptome and proteome. Red circles represent genes upregulated in both transcriptome and proteome; blue circles represent genes downregulated in both; **B** Venn diagram of differential genes under different CCI conditions; **C** KEGG and GO enrichment analysis of commonly downregulated genes; **D** DGSEA analysis graph between pathways; **E** GSEA graph of the pathways with the highest correlation in the DGSEA analysis; **F** The top six significantly downregulated KEGG and GO pathways corresponding to the protein string diagram. Differential genes are arranged from top to bottom by increasing fold change

Although the previous section did not involve virus infection, the rapid decline in protein synthesis capacity from 60 to 84 h during cell culture suggested potential impacts during the 0–24 hpi virus infection phase under CCI3. To directly compare the host cell transcriptomic and proteomic responses triggered by infection under CCI1 and CCI3 conditions, the CCI1 24 hpi and CCI3 24 hpi groups were designated as the experimental groups, with uninfected cell culture samples serving as the control group. Integrative omics analysis was performed to identify reliable differential genes at the transcriptional and protein levels (Fig. 5a). Venn diagram analysis of co-expressed differential genes identified in CCI1 and CCI3 groups (Fig. 5b) and the CCI1 co-expressed downregulated genes were enriched in ribosome, translation (Fig. 5c). The top ten KEGG pathways with the most significant changes at the protein level were identified by GSEA analysis, and their interrelationships were analyzed through DGSEA (Fig. 5d). Results indicated that for CCI1, there was a high correlation between the upregulation of metabolic pathways and downregulation of ribosomal pathways. Excluding cell growth factors, baculovirus infection under CCI1 also downregulated host cell protein synthesis capabilities, similar to other omics studies (Nayyar et al. 2017). Co-expressed differential genes in the CCI3 group were enriched in oxidative phosphorylation, ribosomal biogenesis, and other pathways. DGSEA analysis showed that the highest correlation was between downregulation of oxidative phosphorylation/spliceosome and upregulation of amino acid metabolism/carbon metabolism.

Additionally, infection under CCI3 conditions led to a severe downregulation of mitochondrial-related proteins, with the top four proteins with the largest differential fold: mitochondrial cytochrome b-c1 complex subunit 6 (QCR6, XP_026740563), NADH dehydrogenase [ubiquinone] flavoprotein 1 (NDUFV1, XP_026737816), NADH dehydrogenase [ubiquinone] 1 alpha subcomplex subunit 6 (NDUFA6, XP_026734396), NADH dehydrogenase [ubiquinone] 1 alpha subcomplex subunit 12 (NDUFA12, XP_026742056) (Fig. 5f). QCR6 is a subunit of the cytochrome bc1 complex, an inner membrane protein that catalyzes the oxidation of ubiquinol and the reduction of cytochrome c in the mitochondrial respiratory chain and bacterial photosynthesis and respiration (Crofts 2004). NDUFV1, NDUFA6, and NDUFA12 are subunits of mitochondrial complex I, a complex polymeric membrane-bound complex consisting of 14 conserved catalytic core subunits and up to 31 additional subunits that contribute to its stability, regulation, and biogenesis (Chung et al. 2022). The mitochondrial complex I catalyzes the transfer of electrons from NADH to ubiquinone (Q) and couples this to the translocation of protons across the mitochondrial inner membrane, establishing an electrochemical gradient to drive ATP synthesis. The severe downregulation of these genes during infection may lead to significant mitochondrial dysfunction, further exacerbating the energy burden on host cells during viral infection.

## Cellular senescence and mitochondrial dysfunction *as major* causes of cell density effect

We first verified the hypothesis of senescence in High Five cells cultivation. The characteristics of cellular senescence included changes in cell cycle, the activity of Senescence-associated β-galactosidase (SA-β-gal), oxidative stress levels, and mitochondrial function.

The progression of the cell cycle is strictly controlled by various checkpoints, with the G1/S checkpoint being particularly crucial. At this point, cells integrate and transmit complex internal and external signals such as growth factors, mitogens, and DNA damage, to decide whether to undergo DNA replication, cell division, apoptosis, or enter the G0 phase (Valentijn et al. 2018). In this study, flow cytometry was used to monitor the cell cycle during cell growth (Table 2). The results indicated that the proportion of cells in the G1 phase gradually increased during culture, while the proportion in the S phase gradually decreased. The proportion of cells in the G2 phase reached its lowest value at 36 h and then gradually increased. The increase in the proportions of cells in the G1 and G2 phases during culture suggests that cells may detect DNA damage at the G1/S and G2/M checkpoints, leading to cell cycle arrest to prevent cells with damaged DNA from entering the division phase.

**Table 2:** Cell cycle during High Five cell culture

	G1 (%)	S (%)	G2 (%)
12 h	48.5	17.8	14.3
36 h	51.6	17.6	10.2
60 h	51.8	15.0	13.0
84 h	57.7	8.69	18.2

**Fig. 6:**
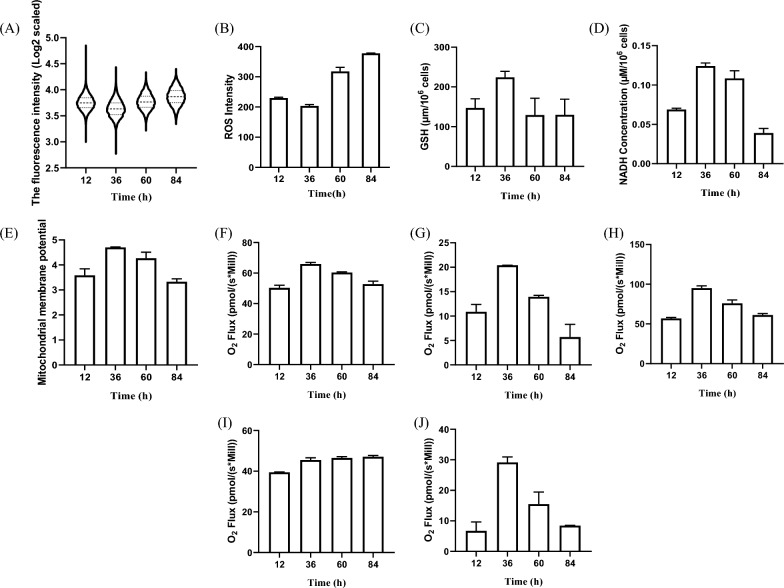
Senescence-related detection during cell culture process. **A** β-galactosidase (SA-β-gal) activity; **B** Intracellular ROS concentration; **C** Intracellular GSH concentration; **D** Intracellular NADH concentration; **E** Mitochondrial membrane potential; **F** Basal respiratory capacity; **G** Proton leak respiration; **H** Maximum respiratory capacity; **I** ATP production capacity; **J** Spare respiratory capacity

SA-β-gal is highly overexpressed in senescent cells and regarded as a marker of senescence. We monitored changes in the SA-β-gal activity using flow cytometry. After entering live cells, the fluorescent β-Gal substrate is cleaved by senescence-associated β-galactosidase. Results showed that the average intensity of green fluorescence gradually increased after 36 h of cell culture (Fig. 6a), and the range between the minimum and maximum values gradually decreased. This indicates that as cell culture progresses, the cell population is gradually advancing towards senescence.

Oxidative damage caused by ROS is one of the major theories of cellular senescence (Chapman et al. 2019). In this study, as the culture progressed, the ROS levels in High Five cells were minimal at 36 h of culture and then rapidly accumulated in the cells (Fig. 6b). Antioxidant capacity analysis showed that the cells had the highest concentrations of GSH and NADH at 36 h of culture. As the culture time increased, the intracellular concentrations of GSH and NADH observably declined (Fig. 6c and d). Mitochondrial dysfunction is a hallmark of senescence. In this study, we monitored mitochondrial membrane potential and respiration capacity during the cell growth process. Results showed that at 36 h, cells exhibited the highest mitochondrial membrane potential (Fig. 6e). At this time, High Five cells had the highest capability of basal respiration, proton leak respiration, maximum respiration (Fig. 6f–h). Notably, High Five cells exhibited a remarkably high SRC value at 36 h of culture (Fig. 6j), calculated as the difference between maximum respiration and basal respiration. After this point, the SRC value rapidly declined. SRC reflects the cell’s ability to produce more ATP by increasing mitochondrial oxidative phosphorylation activity in response to additional energy demands. Low SRC levels may correspond to mitochondrial dysfunction that is difficult to detect under basal conditions. These analyses indicated that High Five cells exhibited characteristics of senescence and mitochondrial dysfunction in the later stages of culture.

## The *ndufa12* gene is a key regulatory gene in the cell density effect

**Fig. 7:**
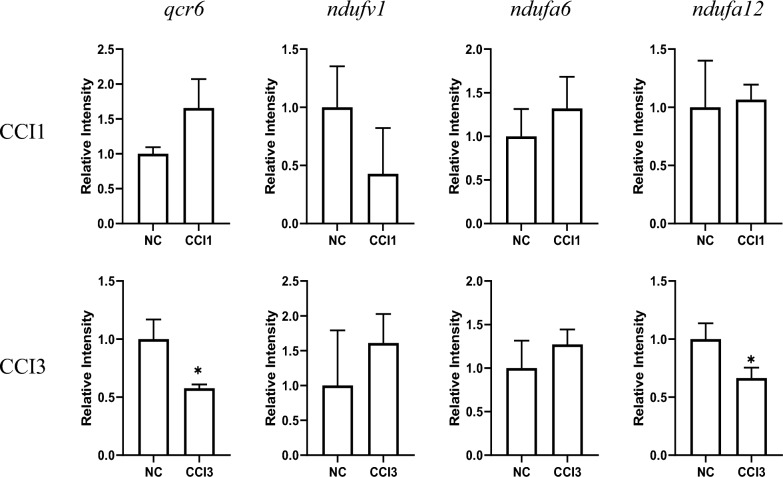
Verification of qPCR transcript levels of four significantly different genes identified by omics

The SRC was hypothesized to be crucial for the productivity of PCV2-VLPs. Therefore, based on the results of transcriptomic and proteomic analyses, RT-qPCR was further employed to detect the changes in mRNA expression levels of four significantly differentially expressed genes related to respiration during the virus infection stage under CCI1 and CCI3 conditions (Fig. 7). Uninfected cell culture samples served as the control group. In the CCI1 group, the *ndufv1* gene was identified as significantly downregulated (*p* < 0.001), while the *ndufa6* gene was identified as significantly upregulated (*p* < 0.01). Although no significant downregulation of *qcr6* and *ndufa12* genes was observed under CCI1, their mRNA expression was significantly inhibited at 24 hpi under CCI3 (*p* < 0.001; *p* < 0.001), with expression levels decreased by 47.4% and 69.4% respectively. The differences in mRNA expression levels of *qcr6* and *ndufa12* genes under different CCI conditions were consistent with the integrated omic analyses, suggesting that these two genes were associated with cell density.

**Fig. 8:**
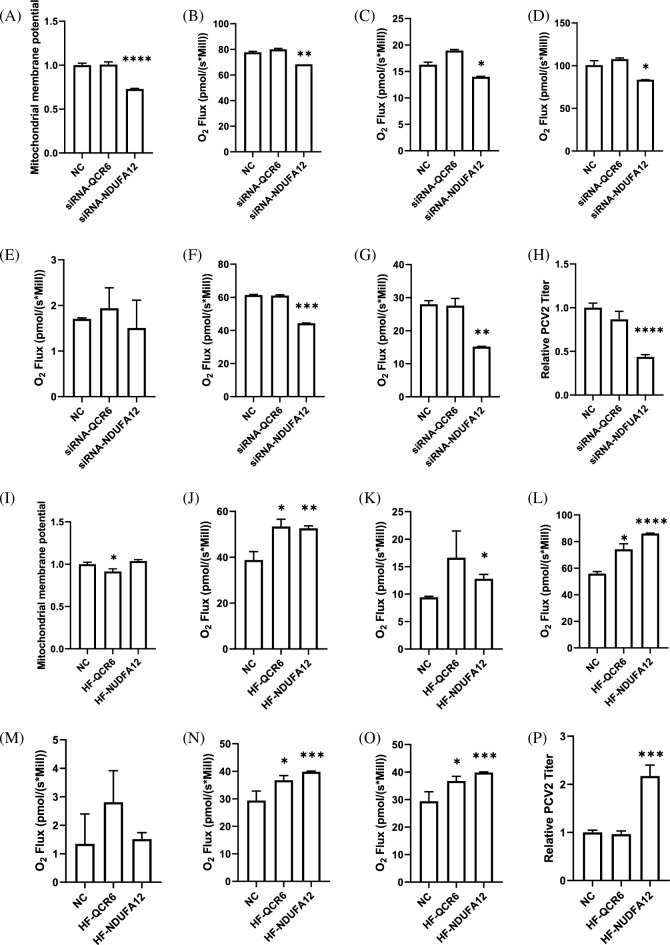
Functional verification of *qcr6* and *ndufa12* genes. siRNA knockdown cell lines at CCI1 0hpi, **A** mitochondrial membrane potential; **B** basal respiratory capacity; **C** proton leak respiratory capacity; **D** maximum respiratory capacity; **E** residual respiratory capacity outside mitochondria; **F** ATP production capacity; **G** spare respiratory capacity; **H** total PCV2 titer at 72hpi. Overexpression cell lines at CCI3 0hpi, **I** mitochondrial membrane potential; **J** basal respiratory capacity; **K** proton leak respiratory capacity; **L** maximum respiratory capacity; **M** residual respiratory capacity outside mitochondria; **N** ATP production capacity; **O** spare respiratory capacity; **P** total PCV2 titer at 72hpi

The siRNA interference technology and recombinant plasmid overexpression technology were utilized to verify the function of *qcr6* and *ndufa12* genes. First, RT-qPCR analysis was used to validate the feasibility of gene knockdown and overexpression at the transcriptional level. The results showed that siRNA interference significantly reduced the mRNA expression levels of *qcr6* and *ndufa12* genes (*p* < 0.001; *p* < 0.01, Fig. S1a and b), while overexpression significantly increased their mRNA expression levels (*p* < 0.001; *p* < 0.01, Fig. S1c and d). At 0 hpi under CCI1, downregulation of the *qcr6* gene did not inhibit mitochondrial membrane potential, respiration capability, and the production capacity of PCV2-VLPs also decreased (Fig. 8). At 0 hpi under CCI3 conditions, overexpression of the *qcr6* gene comprehensively enhanced mitochondrial respiration capacity (Fig. 8j–o), particularly improving the SRC value (*p* < 0.05). These analyses indicated that overexpression of the *qcr6* gene can improve mitochondrial function to some extent, but this gene was not directly related to the “cell density effect”.

At 0 hpi under CCI1, reducing the expression level of the *ndufa12* gene significantly lowered mitochondrial membrane potential (*p* < 0.0001) (Fig. 8a), severely inhibited the basal respiration capacity of High Five cells (*p* < 0.001), proton leak respiration capacity (*p* < 0.05), maximum respiratory capacity (*p* < 0.05), ATP production capacity (*p* < 0.01), and SRC (*p* < 0.01) (Fig. 8b–d, f and g). The production capacity of PCV2 also was significantly inhibited (*p* < 0.0001) (Fig. 8h). The siRNA interference studies indicated that the *ndufa12* gene directly affects normal mitochondrial function and homeostasis, causing healthy cells to exhibit an energy metabolism state similar to that of senescent cells, ultimately inhibiting the PCV2-VLPs production capacity of the cells. At 0 hpi under CCI3, although there was no significant change in mitochondrial membrane potential, overexpression of the *ndufa12* gene comprehensively enhanced mitochondrial respiration capacity, including basal respiration capacity (*p* < 0.05), proton leak respiration capacity (*p* < 0.05), and maximum respiratory capacity (*p* < 0.01) (Fig. 8j–l). It was worth noting that both ATP production capacity and SRC were significantly improved (*p* < 0.001; *p* < 0.001) (Fig. 8n and o). Additionally, overexpression of the *ndufa12* gene significantly improved the PCV2-VLPs production capacity (*p* < 0.001) (Fig. 8p). These studies suggested that overexpression of the *ndufa12* gene can effectively improve mitochondrial function and energy metabolism in high-density cells, partially reversing the senescence trend of the cells, thereby effectively improving the PCV2-VLPs production capacity limited by the “cell density effect” under CCI3 conditions.

## Discussion

The occurrence of the “cell density effect” is a complex process influenced by multiple factors. Although the cell culture process is affected by external factors such as the consumption of nutrients and the accumulation of metabolic by-products, the microfunctional changes induced by the host cells with the density increasing may be the direct factors influencing the production capacity of PCV2-VLPs.

During cell culture, to maintain cell viability as much as possible, fresh culture medium is used to dilute the cell suspension during the exponential growth phase or just before entering the deceleration phase, keeping the cells in a healthy state. The aging phenomenon that may occur during this process is called replicative senescence, which refers to the state where some cells can only undergo a limited number of divisions in vitro before entering an irreversible growth arrest. Another type of aging is known as stress-induced premature senescence (SIPS), characterized by irreversible cell division arrest and the appearance of senescence phenotypes in normal, immortalized, or transformed cells after various types of stress leading to oxidative stress and/or DNA damage (Rattan and Hayflick 2016). Unlike replicative senescence, SIPS typically lacks the feature of telomere shortening (Fahy et al. 2010). During a batch of cell culture, High Five cells undergo only a few divisions. SIPS is highly likely to occur during high-density insect cell cultures in vitro and extend to affect critical periods of PCV2-VLPs subunit vaccine production. SIPS can originate from internal or external sources, primarily including oxidative stress due to mitochondrial degeneration, genotoxic stress due to chromatin damage, and oncogenic stress leading to oncogene-induced aging (Jaggi et al. 2011; Lushchak 2014; Mohamad Kamal et al. 2020). Oxidative stress refers to an imbalance between the production and accumulation of ROS in cells and tissues and the ability of biological system to detoxify these reactive products (Tan et al. 2018). When ROS levels exceed the antioxidant capacity of cells, they cause DNA damage, protein and lipid oxidation, leading to the formation of oxides or peroxides, which in turn cause cell membrane structural damage, changes in permeability, and cytotoxic reactions (Mohamad Kamal et al. 2020).

The success of viral infection highly depends on the metabolic state of the cells at the time of infection and the viral manipulation of energy metabolism to meet these needs (Carinhas et al. 2010). Differences in energy states can even lead to variations in infection efficacy among different host species. Zika virus can infect human peripheral neurons and induce apoptosis, but it does not adversely affect the health of the mosquito vector throughout its lifecycle (Daep et al. 2014). Studies have shown that Zika virus infection in human cells leads to increased AMP/ATP ratios, phosphorylation of AMPK, and caspase-mediated cell death, whereas these phenomena are not observed in Zika virus-infected C6/36 mosquito cells (Thaker et al. 2019). Metabolomics analysis shows that Zika virus infection tends to shift glucose metabolism towards glycolysis and the TCA cycle in human cells, whereas it favors the PPP pathway in C6/36 mosquito cells for survival and glucose metabolism (Thaker et al. 2019). In this study, High Five cells exhibited the optimal energy state under CCI1. After virus inoculation, the host cell downregulated the ribosome's ability to effectively synthesize proteins, reducing the synthesis of the host cell's own proteins and reallocating more resources for viral protein synthesis. On the other hand, the cells upregulated the pentose phosphate pathway (PPP) to provide more nucleotides for viral protein replication. However, at 0 hpi under CCI3, the energy state and spare respiratory capacity were insufficient. To meet the energy demands of viral infection, the cells upregulated genes related to carbon/amino acid metabolism, rather than mobilizing all host cell resources for viral protein replication, which significantly limits the productivity of PCV2-VLPs under CCI3.

Cellular senescence may trigger a decrease in the ability of host cells to resist various stresses. SRC reflects the ability to produce more ATP by increasing mitochondrial oxidative phosphorylation activity when facing additional energy demands (Brand and Nicholls 2011). SRC characterizes the ability of mitochondria to meet additional energy demands beyond basal levels in response to acute cellular stress or heavy workload, thus avoiding an ATP crisis (Marchetti et al. 2020). The low SRC levels may correspond to mitochondrial dysfunction not visible under basal conditions (Brand and Nicholls 2011). SRC is considered a neuroprotective mechanism against excitotoxicity in fibroblasts (DeWaal et al. 2018), and is thought to be related to resistance to hypoxia-induced cell death in cardiac cells (Esteves et al. 2014). SRC depends on the integrity of the mitochondrial electron transport chain and the proton permeability of the mitochondrial inner membrane, the availability of mitochondrial substrates and TCA cycle activity, and mitochondrial homeostasis (Marchetti et al. 2020). In this study, we observed a rapid decline in SRC levels in the later stages of culture, indicating a weakened capacity to respond to stimulation during virus infection. This decline in SRC levels during growth may be caused by aging, greatly deteriorating mitochondrial structure and function (Marchetti et al. 2020). A study of fibroblasts from 55 individuals of different ages showed a sharp decrease in SRC levels, with a 20% decrease in donors over the age of 61 (Greco et al. 2003). In this study, we observed a rapid decline in SRC levels in the later stages of culture. At this time, viral infection may exacerbate the energy burden on host cells and cause severe mitochondrial dysfunction.

In this study, infection at high cell density resulted in severe downregulation of several key genes *qcr6*, *ndufv1*, *ndufa6*, and *ndufa12*. These four key genes are all located in the inner mitochondrial membrane, corresponding to complex I and complex III in the electron transport chain. Among them, the expression levels of *qcr6* and *ndufa12* genes showed obvious cell density dependence in both the cell culture stage and the virus infection stage. Overexpression and siRNA knockdown of the two genes showed that the *ndufa12* gene has an important impact on mitochondrial function and final yield. Overexpression of the *ndufa12* gene can effectively alleviate the cell density effect and may be a potential regulatory gene for delaying cellular senescence. The *ndufa12* gene is considered to play a role in assembling and stabilizing the external arm of mitochondrial respiratory chain complex I (Rak and Rustin 2014). Recent research has emphasized the importance of the *ndufa12* gene, with mutations linked to severe mitochondrial diseases (Magrinelli et al. 2022; Ostergaard et al. 2011; Torraco et al. 2021). Furthermore, NDUFA12 has emerged as a target protein for the natural small molecule Ertredin, which has demonstrated efficacy in inhibiting the growth of cancer cells harboring EGFRvIII mutations, associated with anti-tumor activity (Park et al. 2024). Although parts of mitochondrial complex I, such as NDUFS1, NDUFS8, and NDUFV1, have been extensively studied, the specific functions of other subunits remain largely unexplored, warranting further investigation to uncover novel therapeutic targets as a promising avenue for future research.

In summary, this study highlighted the complex interplay between cell density, mitochondrial function, and cellular senescence, particularly stress-induced premature senescence, in High Five cells during the production of PCV2-VLPs. Our findings indicated that SIPS, driven by oxidative stress and mitochondrial dysfunction, contributed significantly to the cell density effect, limiting viral protein production. We identified the downregulation of key mitochondrial genes, especially *ndufa12*, as a critical factor in this process, suggesting that enhancing the expression of such genes could improve mitochondrial function, delay cellular senescence, and ultimately enhance PCV2-VLPs yield. These insights offered potential strategies for optimizing the BEVS, making it possible to increase efficiency and productivity in both vaccine development and protein engineering.

## Supplementary Information

Additional file 1.

## Data Availability

All data sets used and analyzed are available on reasonable request. The transcriptomics data have been deposited to the GEO repository under accession number GSE280032 (https://www.ncbi.nlm.nih.gov/geo/query/acc.cgi?acc=GSE280032), and the proteomics data are available in the iProX repository, with the dataset identifier IPX0010092000 (https://www.iprox.cn/page/project.html?id=IPX0010092000).
