# Supplementary material for: Stress-induced premature senescence in high five cell cultures: a principal factor in cell-density effects
Source: Bioresour Bioprocess. 2024 Nov 25;11(1):107. doi: 10.1186/s40643-024-00824-x (PMC11589019; doi:10.1186/s40643-024-00824-x)
Supplement: Supplementary file 1 — Additional file 1. [file 40643_2024_824_MOESM1_ESM.docx]

**TITLE**

# Stress-Induced Premature Senescence in High Five Cell Cultures: A Principal Factor in Cell-Density Effects

**AUTHOR**

Rui Min^1^, Dahe Zhang^2^, Mingzhe He^2^, Jingyuan Chen^1^, Xiaoping Yi^1*^, Yingping Zhuang^1^

**AFFILIATION**

1. State Key Laboratory of Bioreactor Engineering, East China University of Science and Technology (ECUST), Shanghai, China

2. Womei Biology Company, Limited, Suzhou, China

** Correspondence author:*

*Xiaoping Yi*

East China University of Science and Technology, 130 Meilong Rd., Shanghai 200237, China. Tel: +86 021 64251131. E-mail: [xpyi@ecust.edu.cn](mailto:xpyi@ecust.edu.cn)

**Table S1** Primer sequences for qPCR.

|  | | **Sense（5'-3'）** | **Antisense（5'-3'）** |
| --- | --- | --- | --- |
| *qcr6* | GGCTGGCAACATTGATTCAC | | CTGTGAGCACACCTCCCTTA |
| *ndufv1* | TGTACAACATCTCGGGGCAC | | CACGGTCTCGCATACGTCTT |
| *ndufa6* | AAGCGCAGAGTATTGGCCTT | | TGAGTTCCATCTGACCCTTGAT |
| *ndufa12* | AGCACTCGACAAAGTCGCAA | | CCATTCTGCTGAAACCTGGC |
| Actin | ATCGCTGACCGTATGCAGAAG | | TTCGAGATCCACATCTGCTGG |

**Table S2** Primer sequences for siRNA analysis.

|  | **Sequences1** | **Sequences2** |
| --- | --- | --- |
| *qcr6* | CAAGUACCAAGAGUGCAAUTT | AUUGCACUCUUGGUACUUGTT |
| *ndufa12* | CGGACUACCACAUGAACUATT | UAGUUCAUGUGGUAGUCCGTT |

**Table S3** Primer sequences for overexpression analysis.

|  | **Sense（5'-3'）** | **Antisense（5'-3'）** |
| --- | --- | --- |
| *qcr6* | TACCGAGCTCGGATCATGTCCAACAAAAACATTATTCCTG | CTGGACTAGTGGATCTTACTTTAGCCTCTTGAAGAGGTCC |
| *ndufa12* | TACCGAGCTCGGATCATGTCGCTAGCGAAGTATTTAGCAC | CTGGACTAGTGGATCTTAGGCCTTGGCCTTGGGTTC |


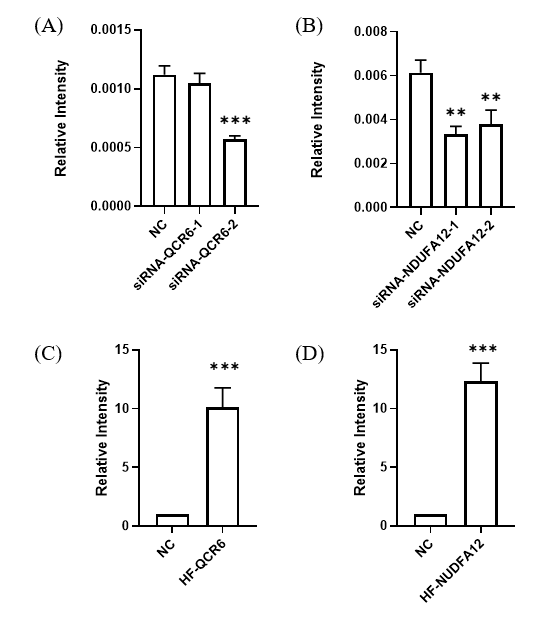


**Fig. S1** qPCR analysis of (A) *qcr6* and (B) *ndufa12* siRNA sequences. Sequences with high interference efficiency were selected for the experiment. qPCR analysis of overexpressing (C) *qcr6* and (D) *ndufa12*.
